# The influence of audience’s regulatory focus on the persuasive effect of different pro-vaccine messages

**DOI:** 10.1371/journal.pone.0328638

**Published:** 2025-08-06

**Authors:** Dengke Xia, Yue Su, Mengyao Song, Tingshao Zhu, Nan Zhao, Hongwei Sun

**Affiliations:** 1 Department of Psychology, Shandong Second Medical University, Weifang, Shandong, China; 2 State Key Laboratory of Cognitive Science and Mental Health, Institute of Psychology, Chinese Academy of Sciences, Beijing, China; 3 Department of Psychology, University of Chinese Academy of Sciences, Beijing, China; Regional Health Care and Social Agency of Lodi, ITALY

## Abstract

The aim of this study was to investigate the influence of an audience’s regulatory focus on the persuasive effect of different pro-vaccine messages. Study 1 (*N* = 169) examined attitude changes among audiences with different regulatory focus after reading pro-vaccine messages, and Study 2 (*N* = 201) added the exploration of message persuasiveness and vaccination intentions as well. The results of Study 1 and Study 2 showed that both high promotion fucus and high prevention focus could facilitate the attitude change elicited by messages emphasizing *vaccine safety*, *official position* and *credibility*. Meanwhile, high-promotion individuals were also more easily influenced by messages highlighting the benefits of vaccination, while high-prevention individuals were more receptive to messages focusing on the personal responsibility. Similar results also appeared when measuring perceived persuasiveness as dependent variable. However, these impacts of regulatory focus did not remain statistically significant on behavioral intention for most messages, except for the messages emphasizing *vaccine effectiveness*. The behavioral intention changes after reading these messages were significantly promoted by high promotion focus. Our findings could shed some light on the mechanism underlying personalized persuasion as well as more targeted vaccination promotion strategies.

## Introduction

Vaccination is widely acknowledged as the most effective way to respond to pandemics by combating disease infection and reducing mortality rates [1]. People can shield themselves from diseases and reduce their chances of becoming disease vectors by getting vaccinated [2]. During COVID-19 pandemic, vaccination is considered as one of the most effective methods to control the pandemic [3]. However, widespread hesitancy towards vaccines has limited their effectiveness in addressing pandemic issues [4].

In the digital age, online platforms have become the primary means of promoting vaccination by releasing related messages to persuade the public to be vaccinated. Various studies have demonstrated that increased exposure to those information can raise public’s awareness of vaccines, thereby promoting their willingness to vaccinate [5]. The publication of explanatory messages regarding the benefits in achieving herd immunity has played a crucial role in promoting vaccination [2].

The content of pro-vaccine messages can significantly influence their persuasive effect of vaccination: narrative messages can raise awareness of influenza risk and vaccine effectiveness and promote vaccination [6], humorous messages can raise awareness of pandemic severity and reduce vaccine hesitation [7], and the framing or rhetoric of the message can also have an impact on persuasion [8]. Meanwhile, individual’s personality traits could also influence their responses to pro-vaccine information [9]. One of the key characteristic is regulatory focus, which has been found to have significant impacts on the processes of persuasion [10].

As a personality trait theory, Regulatory Focus Theory (RFT) categorizes individual’s traits into two motivational systems: promotion and prevention [11]. High-promotion individuals perceive goals as ideals. They are sensitive to positive outcomes and motivated to pursue gains, prioritizing growth and achievement. In contrast, high-prevention individuals view goals as obligations. They are sensitive to negative outcomes and motivated to avoid losses, prioritizing security and responsibility [12–14]. People with different regulatory focus tend to use different goal-pursuit strategies [15]. High promotion individuals adopt eager strategies driven by growth, while high prevention individuals use vigilant strategies driven by safety [16]. Compared to promotion-focused individuals, those with a prevention focus are more sensitive to losses and display greater conservatism in judgment and decision-making [17].

Since regulatory focus directly influences individuals’ assessment of benefits and risks, it plays a crucial role in persuading them to take actions involving potential gains or losses (e.g., taking medication on time, getting vaccinated) [17,18]. Previous studies have highlighted its importance in persuasion, showing that the effectiveness of different message frames could be affected by the audience’s regulatory focus [10,19]. Depending on the target states described, the frames could be divided into “gain framed” and “lose framed”. The former emphasizes achieving positive outcomes or avoiding negative outcomes, while the latter highlights bearing negative outcomes or failing to attain positives [20–22]. A sense of regulatory fit occurs when the frame aligns with the product’s or the audience’s regulatory focus and enhance persuasion [23]. For example, ads warning that not taking a drug leads to clogged arteries are more persuasive than those stating that taking the drug prevents blockage [24]. High-promotion individuals are more motivated by content featuring positive role models and goal achievement [25]. However, prior studies have largely focused on message framing and regulatory fit, neglecting other content dimensions such as topical focus and persuasive strategies. To design more effective pro-vaccine communication and increase uptake, it is essential to understand how different message components interact with personality traits like regulatory focus [26].

To examine the effectiveness of different pro-vaccine messages content on different personality audiences, COVID-19 vaccination serves as a suitable case for conducting such study. As a population-wide pandemic, COVID-19 required nearly everyone, not just high-risk groups, to make vaccination decisions, which reduced confounding effects from differences in personal involvement. Additionally, the vaccine was free in many countries, including our study context, minimizing economic influences and allowing us to focus on communication effects. Prior research has identified the key factors influencing individuals’ COVID-19 vaccination decisions [27], which we could use to structure message content. In this study, we investigated how audience regulatory focus moderated the persuasive effectiveness of these factors. Since the vaccination attitude is a crucial predictor of vaccination [28,29], we chose attitude as the indicator of persuasive effectiveness. Therefore, we propose the following hypothesis.

**H**_**01**_: The regulatory focus of individuals has no impact on their attitude changes after reading the pro-vaccine messages with some influencing factors.
**H**_**11**_: The regulatory focus of individuals can impact their attitude changes after reading the pro-vaccine messages with some influencing factors.

## Study 1

### Methods

#### Ethics statement.

This study received ethical approval from the Institutional Review Board of the Institute of Psychology, Chinese Academy of Sciences with the ethics approval number H23089. All subjects participating in the study were adults and provided informed consent through a standardized electronic process, which included: (1) study information (e.g., purpose, voluntary participation, withdrawal rights, and confidentiality procedures), (2) explicit confirmation of the agreement (“I have read and agree to participate”), and (3) binary continuation option (proceed/exit). Only consenting adults were permitted to complete the online questionnaire.

#### Participants.

The current study was conducted in the contextualized background of a COVID-19 pandemic. We conducted data collection through the online questionnaire platform *Wenjuan Xing* from 20/01/2024, to 27/01/2024, and published our participant recruitment advertisement on Sina Weibo, the most popular social media in China. The inclusion criteria for participants were as follows: Chinese citizens who resided in mainland China during the pandemic and were aged 18 years or older. The exclusion criteria included disabilities that might hinder the completion of the questionnaire and any contraindications to COVID-19 vaccination. We finally obtained 169 valid participants in the collection (M_age _= 24.19, *SD* = 6.73, 46.7% male). Participants were required to report their COVID-19 vaccination status, and all of them indicated having completed the full course of vaccination. This was consistent with the high vaccination rates reported in mainland China in 2023 (over 92.9% [30]).

### Materials and measurements

#### Preparation of pro-vaccine messages and measurement of attitude changes.

A questionnaire was employed to measure the extent of the subjects’ attitude changes after reading the pro-vaccine messages with different influencing factors. These factors were involved in COVID-19 vaccination decision in the study by Su and colleagues [27]. Su et al. (2023) used semi-structured interviews to gain insight into public concerns and motivations regarding COVID-19 vaccination, and identified 20 influencing factors, including *vaccine safety, vaccine effectiveness,* and others (Table 1). And based on the definitions of the factors, we chose three COVID-19 pro-vaccine messages representing each factor from *Sina Weibo*, the most popular Chinese social media. We invited three graduate students in psychology who studying persuasion to evaluate the representativeness of each text on a 7-point scale (*slightly representative–extremely representative*), drawing on Thomas et al.’ s methodology of message validation [31]. Higher scores indicated that the message could better represent the specific influencing factor. In addition, raters were required to determine whether additional influencing factors were also represented in the texts and rating the degree of them. Then we modified those that represented multiple influencing factors, usually by directly dropping a part of the message, had them rated again to ensure that each one contained only one influencing factor. For example:

**Table 1:** The name and definition of each influencing factor.

No.	Influencing factor	Operational definition
1	*Vaccine safety*	The vaccine does not pose a threat to personal safety, such as a low incidence rate of serious untoward effect and the good health condition of population after vaccination.
2	*Vaccination restriction and contraindication*	The message specifies the range of specific people who are not suitable for vaccination or contraindications.
3	*Untoward effect*	The message provides a clear and unambiguous description of the specifics of the adverse reaction and does not contain arbitrary, omitted or ambiguous expressions.
4	*Vaccine effectiveness*	The effective result and effective time period. That is, the vaccine could protect individuals from the infection of corona virus with a high probability, and the vaccine is effective in a specific period of time.
5	*Credibility*	Trustworthiness means message receivers believe that the communicator is able and willing to provide objective, fair, true and effective message. Communicator should be sincere, honest and objective, and have no specific communication motives and intentions.
6	*Official position*	The publisher of the message has a government background, such as the government or other official media.
7	*Vaccination certification*	The message reflects the difference that the vaccination of an individual brings to the individual, such as the color change of the QR code.
8	*Unofficial position*	The publisher of the message is not the official medium, but some media with a neutral political position and no political purpose.
9	*Physical benefit*	The message reflects other benign reactions to the body that are not related to the effects of the vaccine itself, such as weight loss, after the individual has received the vaccine.
10	*Attraction*	Attraction means we are more likely to follow a request from someone we admire or like.
11	*Risk perception*	Individuals’ subjective judgments about how safe their current environment is, how serious the pandemic is, and how much their risk of infection is.
12	*Responsibility*	The message reflects the individual’s awareness, feelings and beliefs about responsibility for oneself and others, for the family and the collective, for the state and society, as well as the corresponding conscious attitude of observing norms, taking responsibility and fulfilling obligations.
13	*High standard group*	The message reflects the use of other out-groups as references when individuals adopt a certain behavior or form a certain attitude. These groups usually have the value of benchmarking and imitation.
14	*Surrounding groups*	The message reflects the fact that when an individual takes a certain behavior, it is influenced by the people around the individual or who have the same background as the individual’s life.
15	*Dual role persuasion*	Both positive and negative aspects are provided in the message.
16	*Ingroup pressure*	The message reflects the fact that individuals are influenced by the group they are in and need to conform to group opinions or norms in order to maintain a relationship with the group.
17	*Social benefits*	The message reflects the social or life benefits or conveniences of individual vaccination that are not related to the effects of the vaccine itself, such as ease of travel, vaccination incentives, etc.
18	*Conformity*	The message reflects that vaccination and the behaviors and perceptions that promote it are the choice of most people, and are something that people are generally willing to do.
19	*Obey*	The message reflects an individual’s behavior that is compelled by social demands, group norms, or the will of others to adhere to conform to the demands of others or norms.
20	*Interpretability–External attribution*	Explanations in the information for the relevant behaviors and requirements in the process of vaccine development, vaccination, and promotion, such as the reasons for the specific unsuitability of the unsuitable population for vaccination.

a. Original message: “*The COVID-19 pandemic won’t go away. Getting vaccinated is not just for yourself, but also for your family and other individuals. Plus, there haven’t been any safety problems reported with the vaccine, so it’s safe and totally nothing to stress over.*” (representing *responsibility* and also *vaccine safety*)
b. Modified message: “*The COVID-19 pandemic won’t go away. Getting vaccinated is not just for yourself, but also for your family and other individuals.*” (representing *responsibility* only)

The Kendall-*W* of the three raters achieved 0.71 (*p *= 0.003), and the results showed that 80% of the messages had an average score of 6 or higher, demonstrating they are highly representative of the corresponding influencing factors.

In the formal experiment, we ultimately kept the most representative message for each influencing factor, and all these messages had a representativeness score above 6. In addition, we constructed a “blank” message without any influencing factor as a control condition (“We should get COVID-19 vaccine”). Each message was presented alongside an item to ask about the subject’s attitude changes on an 11-point Likert scale: “If you had not yet received the full vaccination during the COVID-19 pandemic, how much did your attitude toward getting vaccinated change after reading the message above?” (0 = *No change*, 10 = *Become much more positive*).

#### Regulatory Focus Questionnaire (RFQ).

The present study utilized the Chinese version of the Regulatory Focus Questionnaire (RFQ), which was originally developed by Higgins et al. [32] and later revised by Chinese scholars Yao et al. [33] to assess individual personality traits. The revised RFQ includes 10 items that are divided into two distinct subscales, the Promotion Focus subscale and the Prevention Focus subscale. The Promotion Focus subscale comprises 6 items that inquire about participants’ experiences related to success, such as “I become more motivated to work hard when I achieve something.” The Prevention Focus subscale includes 4 items that ask participants about their experiences related to failure, such as “I often feel disappointed when I fail to achieve important goals.” Responses were rated on a 5-point Likert scale, with “1” indicating “*strongly disagree*” and “5” indicating “*strongly agree*”. A higher score on each subscale indicates a more pronounced personality trait corresponding to that subscale.

### Demographic measurement

Participants were asked to provide their age, gender (1 = male, 2 = female), and education level (1 = primary school, 2 = high school (including technical secondary school), 3 = college (three-year or two-year college diploma), 4 = bachelors, 5 = masters and above). Note that the analyses reported below were conducted both with and without controlling for the three demographic variables. Conclusions drawn from the analyses did not change regardless of whether or not the demographics were controlled for. Consequently, analyses reported below do not covary out the demographics.

### Data collection

All experimental procedures were conducted with the informed consent of the participants. Participants first read the messages about the vaccine, reported attitude changes caused by each message, then completed the Chinese version of the RFQ, and finally provided demographic information.

### Analytical method

Given that promotion focus and prevention focus are two separate dimensions [32], we performed separate analyses as previous researchers did [32]. We calculated the score of each dimension, and classified the top 27% of participants into a high grouping and the lowest 27% into a low grouping for each dimension, as a widely-adopted statistical processing [34,35], in order to better capture the distinctions between the high and low groups. Finally, we constructed high and low grouping for both promotion focus (high promotion: n = 51; low promotion: n = 52) and prevention focus (high prevention: n = 47; low prevention: n = 48).

For each influencing factor, the message containing the factor was designated as the specific-influencing-factor condition and the message without any factor was designated as the control condition. To examine the effects of regulatory focus on the vaccine persuasion when the message content delivering specific influencing factor, we conducted a 2 (regulatory focus [between-subject]: high grouping vs. low grouping) **×** 2 (message content [within-subject]: specific-influencing-factor condition vs. control condition) repeated measures ANOVA for each of the 20 influencing factors. All these analyses were conducted using SPSS 27.

## Results and discussion

### The main effect of regulatory focus and influencing factors

The results of the ANOVA showed that the main effect of regulation focus on attitude change was not statistically significant for the messages with vast majority of influential factors. Specifically, the main effect of promotion focus was not significant for all factors, and the main effect of prevention focus was significant only for *social benefits* (*F* = 8.45, *p* < 0.01, η_p_^2^ = 0.08). The main effects of influencing factors on attitude change showed that for the majority of influencing factors, their persuasive effects were significantly higher than the “blank” message with no specific influencing factor. (see Appendix for details).

### Interaction effect of regulatory focus and influencing factors on attitude change

Interactions were observed between regulatory focus and some influencing factors on attitude change. Specifically, there were significant interactions between promotion focus and *vaccine safety*, *vaccination restrictions and contraindications*, *untoward effect*, *credibility*, *official position*, and *attraction*. And there were significant interactions between prevention focus and *vaccine safety*, *credibility*, *official position*, and *risk perception* (Table 2).

**Table 2:** The significant interactions between regulatory focus and influencing factors on attitude change in Study 1.

Interactions	*F*	95% CI	η_p_^2^	*p*
Promotion **×** Vaccine safety	6.72	[0.36, 2.72]	0.06	0.011
Promotion **×** Vaccination restriction and contraindication	4.07	[0.02, 2.55]	0.04	0.046
Promotion **×** Untoward effect	5.31	[0.20, 2.65]	0.05	0.023
Promotion **×** Credibility	5.77	[0.22, 2.35]	0.05	0.018
Promotion **×** Official position	4.70	[0.10, 2.33]	0.04	0.032
Promotion **×** Attraction	4.49	[-1.89, -0.06]	0.04	0.037
Prevention **×** Vaccine safety	4.12	[0.03, 2.44]	0.04	0.045
Prevention **×** Credibility	6.57	[0.29, 2.32]	0.07	0.012
Prevention **×** Official position	5.74	[0.23, 2.42]	0.06	0.019
Prevention **×** Risk perception	7.07	[0.34, 2.36]	0.07	0.009

*Note*: Promotion (high vs. low) and prevention (high vs. low) are two independent dimensions of regulatory focus, with their interaction effects tested separately against each influencing factor. Methodological details apply to subsequent tables unless noted otherwise.

**Fig 1:**
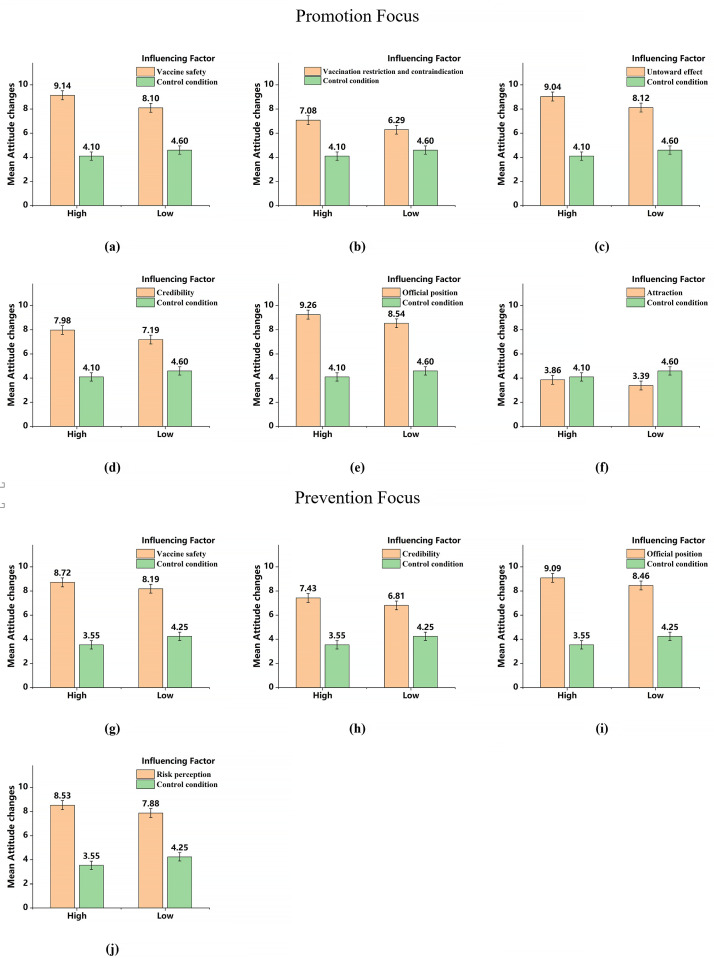
The interaction of regulatory focus and influencing factors on attitude change in Study 1. In a nutshell, we confirmed Hypothesis 1 that individuals’ regulatory focus can influence their attitude change when reading messages with some vaccine-influencing factors. Individuals with a high promotion focus and high prevention focus showed a greater shift toward positive vaccination attitudes after reading information about *vaccine safety*, *credibility*, and *official position*.

The results of the simple effects analysis on the attitude change were shown in Fig 1. As the message with *attraction* (f) cannot induce attitude change higher than a “blank” message, this influencing factor seems not applicable in vaccine persuasion and would not be discussed further. The messages with *vaccine safety* (a), *vaccination restriction and contraindication* (b), *untoward effect* (c), *credibility* (d) and *official position* (e) can enhance the pro-vaccination attitudes in high-promotion individuals to a significantly greater extent than in low-promotion individuals. It is suggested that the high-promotion individuals were more susceptible to the pro-vaccine messages with *vaccine safety*, *vaccination restriction and contraindication*, *untoward effect*, *credibility* and *official position*. Meanwhile, the messages with *vaccine safety* (g), *credibility* (h), *official position* (i) and *risk perception* (j) can enhance the pro-vaccine attitudes in high-prevention individuals to a significantly greater extent than in low-prevention individuals. It is suggested that the high-prevention individuals were more susceptible to the pro-vaccine messages with *vaccine safety*, *credibility*, *official position* and *risk perception*.

Besides attitude change, the effect of message persuasion could be reflected in some different indices, such as perceived persuasiveness and behavior. The persuasiveness of messages can precipitate individuals’ attitude changes after reading. This perception plays a key role in the persuasion process and may lead to attitude changes [36,37]. Behavior change is another significant dimensional manifestation of the persuasion effectiveness. Pro-vaccine behavior directly leads to an increase in vaccination rates, making this indicator highly significant for vaccine persuasion [38]. However, research has shown that attitude change does not always lead to behavior change [38]. To this moment, it remains to be seen whether the audience’s regulatory focus also influence their perception of a message’s persuasiveness or their behavioral tendency of getting vaccinated [39]. In order to probe the effects of regulatory focus in persuasion in more depth, in Study 2 we employed measures assessing participants’ perceptions of the persuasiveness, as well as their willingness to change their behavior after reading the message. The hypothesis of Study 2 are as follows:

**H**_**02**_: The regulatory focus of individuals has no significant impact on their perception of the persuasiveness of pro-vaccine messages, regardless of other influencing factors.
**H**_**12**_: The regulatory focus of individuals can impact their perception of persuasiveness of the pro-vaccine messages with some influencing factors.
**H**_**03**_: The regulatory focus of individuals has no significant impact their intentions of get vaccinated after reading the pro-vaccine messages with some influencing factors.
**H**_**13**_: The regulatory focus of individuals can impact their intentions of get vaccinated after reading the pro-vaccine messages with some influencing factors.

## Study 2

### Methods

#### Participants.

According to G⁎Power we need 102 participants to have an 80% probability of detecting a medium effect (f = 0.2) when conducting a repeated measures, within-between interaction (the effect size estimate was medium effect on the basis of the results from Study 1). To detect the differences between individuals with high and low regulatory focus more effectively, we ultimately recruited 201 participants (between 05/04/2024, and 15/04/2024) using the same methodology as Study 1, comprising 90 males and 111 females, with a mean age of 32.87 (*SD* = 6.415). These participants were also found to be fully vaccinated.

### Materials and measurements

In Study 2, the persuasion messages were prepared through a similar methodology to the one employed in Study 1, and with some modifications. To avoid possible bias due to the random nature of the real messages, we increased the number of representative messages for each influencing factor to three. Similar to Study 1, we selected messages from *Sina Weibo* that could represent each influencing factor based on the definition of the factor, and then invited seven psychologists to rate the representativeness of each message (Kendall-*W *= 0.749, *p *< 0.001). We modified underrepresented messages and finally obtained 60 persuasion messages covering the 20 factors (all messages had a representing score above 6) and 1 control message same as Study 1 (“We should get COVID-19 vaccine”).

In addition, to provide a more complete measure of possible attitude changes of participants, we replaced the unidirectional scale in Study 1 with a 9-point Likert scale with 5 as the midpoint (1 = *Become much more negative,* 5 = *No change*, 9 = *Become much more positive*). We added the item measuring the perceived persuasiveness, “I think this message is persuasive in promoting the COVID-19 vaccination during the pandemic” (1 = *Strongly disagree*, 5 = *Uncertainly*, 9 = *Strongly agree*), and the item measuring the behavioral intention change, “If you had not yet received the full vaccination during the COVID-19 pandemic, how much could read this message change the likelihood of you getting the vaccination?” (1 = *Extremely reduce*, 5 = *Nothing change*, 9 = *Extremely raise*).

### Data collection

After informed consent, participants first read the vaccination-persuasion message, reported their perceived persuasiveness, attitude change and behavioral intention change, then completed the Chinese version of the RFQ, and finally pro-vided demographic information. Given that promotion focus and prevention focus are two separate dimensions [32], we performed separate analyses through SPSS 27.

## Results and discussion

### The main effect of regulatory focus and influencing factors

We employed a similar grouping method as in Study 1, classified the top 27% of participants into a high grouping and the lowest 27% into a low grouping for the promotion focus (High: n = 56, Low: n = 59) and prevention focus (High: n = 63, Low: n = 53). To examine the effects of regulatory focus on the vaccine persuasion when the message content delivering specific vaccine-influencing factors, we conducted repeated-measures ANOVA similar to Study 1. The results showed that the main effect of regulation focus on attitude change, persuasiveness and behavioral intention change was not significant for the vast majority of factors. Specifically, the main effect of promotion focus was significant for the messages with *vaccine safety* (*F* = 4.96, *p* < 0.028, η_p_2 = 0.04), *vaccine effectiveness* (*F* = 6.05, *p* < 0.015, η_p_2 = 0.05), *credibility* (*F* = 4.86, *p* < 0.029, η_p_2 = 0.04) and *official position* (*F* = 4.10, *p* < 0.045, η_p_2 = 0.04) on eliciting behavioral intention change. This suggested that high-promotion focused individuals may be more likely to be induced to get vaccinated after reading pro-vaccine messages sometimes. There was no main effect found among prevention focus.

The main effects of influencing factors on attitude change, perceived persuasiveness and behavioral intention change showed that for the majority of influencing factors, their persuasive effects were significantly higher than the “blank” message with no specific influencing factor. (see Appendix for details).

### The interactions of regulatory focus and influencing factors on attitude change

On attitude change, the results of Study 2 were generally consistent with Study 1 (Table 3). Specifically, there were significant interactions between promotion focus and *vaccine safety*, *vaccination restrictions and contraindications*, *untoward effect*, *credibility*, *official position*, *high standard group* and *attraction*, and there were significant interactions between prevention focus and *vaccine safety*, *responsibility* and *attraction*. Additionally, there were borderline significant interactions between prevention focus and *credibilit*y and *official position*.

**Table 3:** The significant and marginally significant interactions between regulatory focus and influencing factors on attitude change in Study 2.

Interactions	*F*	95% CI	η_p_^2^	*p*
Promotion **×** Vaccine safety^*^	4.20	[0.02, 1.25]	0.04	0.043
Promotion **×** Vaccination restriction and contraindication^*^	3.96	[0.00, 1.17]	0.03	0.049
Promotion **×** Untoward effect^*^	4.34	[0.03, 1.34]	0.04	0.040
Promotion **×** Vaccine effectiveness	5.94	[0.14, 1.38]	0.05	0.016
Promotion **×** Credibility^*^	7.31	[0.24, 1.58]	0.06	0.008
Promotion **×** Official position^*^	6.93	[0.20, 1.40]	0.06	0.010
Promotion **×** Risk perception	4.14	[0.02, 1.36]	0.04	0.044
Promotion **×** Attraction^*^	4.23	[-1.22, -0.02]	0.04	0.042
Prevention **×** Vaccine safety^*^	4.40	[0.04, 1.24]	0.04	0.038
Prevention **×** Credibility^*^	3.11	[-0.08, 1.30]	0.03	0.080
Prevention **×** Official position^*^	2.97	[-0.08, 1.12]	0.03	0.087
Prevention **×** Responsibility	4.28	[0.03, 1.26]	0.04	0.041
Prevention **×** Attraction	4.43	[-1.17, -0.04]	0.04	0.038

*Note*:

* Represents the significant interactions that also appeared in Study 1.

There were also some differences between the results of Study 2 and Study 1. Promotion focus showed new interactions with *vaccine effectiveness* and *risk perception*, while prevention focus showed a new interaction with *responsibility*. In addition, the interaction between prevention focus and *risk perception* did not reappear in Study 2. Such might be attributed to the waning impact of COVID-19 when Study 2 conducted, which turns high-promotion individuals less sensitive to the information about *risk perception*.

**Fig 2:**
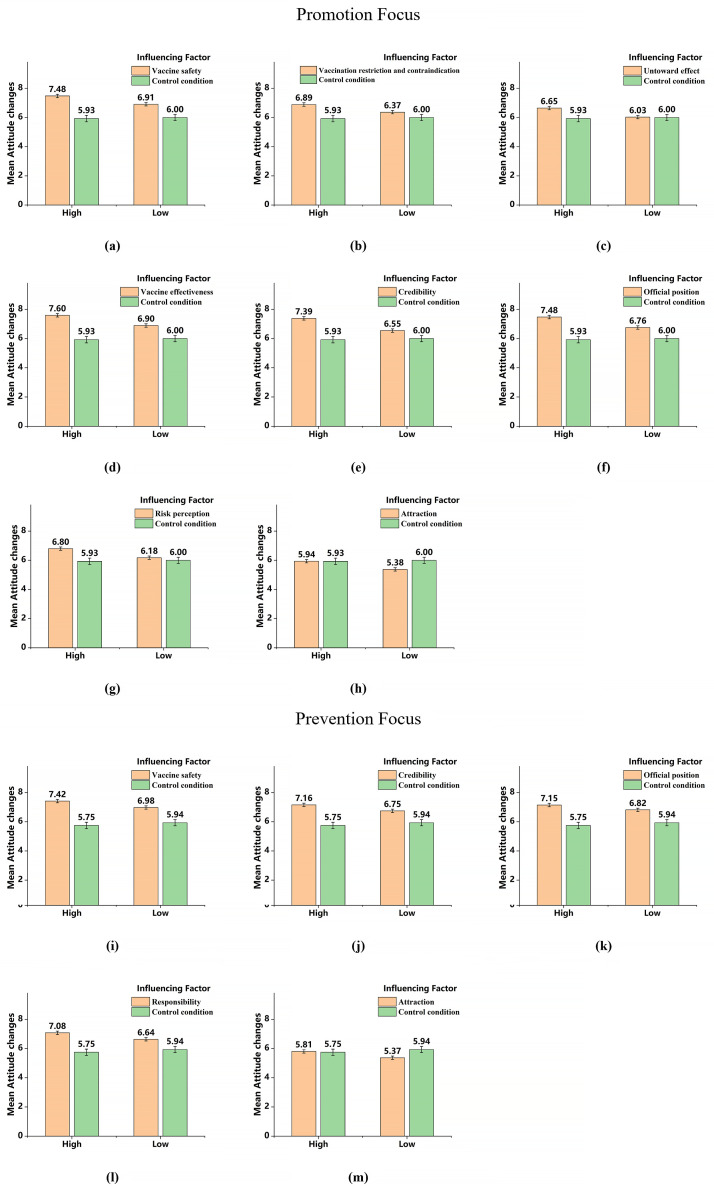
The interaction of regulatory focus and influencing factors on attitude change in Study 2.

The results of the simple effects analyses were shown in Fig 2. Similar to Study 1, the scores of *attraction* (h)/(m) were still lower than control group, and would not be discussed further. The messages with *vaccine safety* (a), *vaccination restriction and contraindication* (b), *untoward effect* (c), *vaccine effectiveness* (d), *credibility* (e), *official position* (f) and *risk perception* (g) can enhance the pro-vaccine attitudes in high-promotion individuals to a significantly greater extent than in low-promotion individuals. Moreover, *vaccine safety* (i), *credibility* (j), *official position* (k) and *responsibility* (l) messages can enhance the pro-vaccine attitudes in high-prevention individuals to a significantly greater extent than in low-prevention individuals.

### The interactions of regulatory focus and influencing factors on persuasiveness and behavioral intention change

We also found interactions between regulatory focus and some influencing factors on perceived persuasiveness and behavioral intention change. Regarding persuasiveness, there were significant interactions between promotion focus and *vaccine safety*, *vaccination restrictions and contraindications*, *untoward effect*, *vaccine effectiveness*, *credibility*, *official position*, *risk perception*, *high standard group* and *attraction*. And there were significant interactions between prevention focus and *vaccine effectiveness*, *official position*, *responsibility*, *interpretability–external attribution* and *attraction* (Table 4). Moreover, there were significant interactions between promotion focus and *vaccine effectiveness* and *ingroup pressure* on behavioral intention change (Table 5).

**Table 4:** The interactions of regulatory focus and influencing factors on persuasiveness.

Interactions	*F*	95% CI	η_p_^2^	*p*
Promotion **×** Vaccine safety	7.60	[0.31, 1.89]	0.06	0.007
Promotion **×** Vaccination restriction and contraindication	5.00	[0.11, 1.77]	0.04	0.027
Promotion **×** Untoward effect	6.76	[0.27, 1.93]	0.06	0.011
Promotion **×** Vaccine effectiveness	6.80	[0.27, 1.94]	0.06	0.010
Promotion **×** Credibility	8.20	[0.36, 1.96]	0.07	0.005
Promotion **×** Official position	5.45	[0.14, 1.66]	0.05	0.021
Promotion **×** Risk perception	4.06	[0.01, 1.57]	0.04	0.046
Promotion **×** High standard group	4.17	[0.02, 1.52]	0.04	0.043
Promotion **×** Attraction	5.13	[-1.50, -0.10]	0.04	0.025
Prevention **×** Vaccine effectiveness	5.25	[0.13, 1.73]	0.04	0.024
Prevention **×** Official position	4.95	[0.09, 1.59]	0.04	0.028
Prevention **× ** Responsibility	4.68	[0.07, 1.58]	0.04	0.033
Promotion **×** Interpretability–External attribution	4.36	[0.04, 1.52[	0.04	0.039
Promotion **×** Attraction	4.53	[-1.41, -0.05]	0.04	0.035

**Table 5:** The interactions of regulatory focus and influencing factors on behavioral intention change.

Interactions	*F*	95% CI	η_p_^2^	*p*
Promotion **×** Vaccine effectiveness	4.82	[0.07, 1.32]	0.04	0.030
Promotion **×** Ingroup pressure	4.64	[-1.42, -0.06]	0.04	0.033

**Fig 3:**
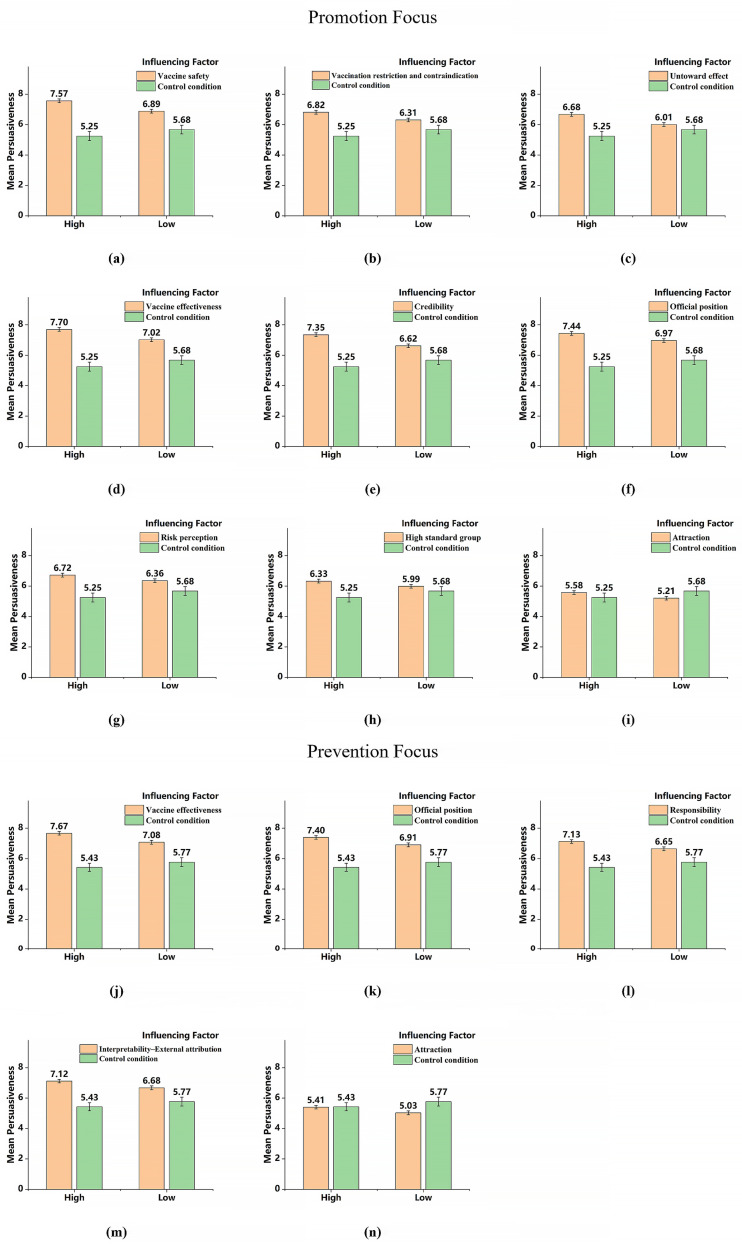
The interaction of regulatory focus and influencing factors on persuasiveness. The results of the simple effect analysis on the behavioral intention change were shown in Fig 4. The messages with *vaccine effectiveness* (a) can enhance the vaccination intention in high-promotion individuals to a significantly greater extent than in low-promotion individuals. It is suggested that the high-promotion individuals were induced to have higher possibilities of get vaccinated than low-promotion individuals, through the messages with *vaccine effectiveness*, which confirmed H_13_. The behavioral intention changes after reading *ingroup pressur*e (b) messages was lower than reading the “blank” message, which means that this interaction does not make much practical sense and need not be discussed further.

The results of the simple effects analysis on the persuasiveness were shown in Fig 3. The scores of *attraction* (i)/(n) were also lower than control group, and would not be discussed further. The messages with *vaccine safety* (a), *vaccination restriction and contraindication* (b), *untoward effect* (c), *vaccine effectiveness* (d), *credibility* (e), *official position* (f), *risk perception* (g) and *high standard group* (h) can enhance perceived persuasiveness in high-promotion individuals to a significantly greater extent than in low-promotion individuals. It is suggested that the high-promotion individuals were more susceptible to the pro-vaccine messages with *vaccine safety*, *vaccination restriction and contraindication*, *untoward effect*, *vaccine effectiveness*, *credibility*, *official position*, *risk perception* and *high standard group.* Moreover, the messages with *vaccine effectiveness* (j), *official position* (k), *responsibility* (l) and *interpretability–external attribution* (m) can enhance perceived persuasiveness in high-prevention individuals to significantly a greater extent than in low-promotion individuals. It is suggested that the high-prevention individuals were more susceptible to the pro-vaccine messages with *vaccine effectiveness*, *official position*, *responsibility* and *interpretability–external attribution*. The H_12_ was confirmed based on the above results.

**Fig 4:**
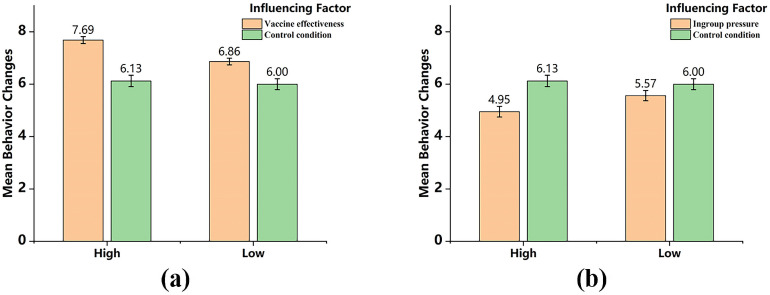
The interaction of regulatory focus and influencing factors on behavior change.

### Analyzing demographics

We calculated bivariate correlations among the demographics and dependent measures. Participant age was negatively associated with the perceived persuasiveness of *physical benefit* (*r*(201) = −0.15, *p* = 0.04), and positively associated with the impact of *vaccine effectiveness* on attitude change (*r*(201) = 0.14, *p* = 0.05). Moreover, education level was negatively associated with the influence of *untoward effect* on persuasiveness (*r*(201) = −0.15, *p* = 0.03), attitude change (*r*(201) = −0.17, *p* < 0.01) and behavioral intention change (*r*(201) = −0.15, *p* = 0.03). To control for the effects of demographic variables on the dependent measures, ANCOVAs were conducted to covary out the effects of gender, age, and education level. Covarying out these confounding variables did not alter the significance of any effects presented above.

### General discussion

We conducted two studies in the COVID-19 scenario to explore how the audience’s regulatory focus influences the persuasion effectiveness of pro-vaccine messages with different influencing factors. Study 1 found the impact of audience’s regulatory focus on the attitude change toward vaccination after reading messages, and Study 2 further revealed whether this impact also appeared on the perceived persuasiveness and the behavioral intention of getting vaccinated, shedding light on the persuasion susceptibility of audiences with disparate regulatory focuses and the mental processes through which persuasion occurred.

The results of both studies indicated that the audience’s regulatory focus could influence the attitude change triggered by the messages with certain vaccination-influencing factor. The attitude of high-promotion individuals were more affected by the messages inherently built in constructs of *vaccine safety*, *vaccination restriction and contraindication*, and *untoward effect*, which are all of high relevance regarding vaccine safety: *Vaccine safety* as a positive cue for safety can distinctly evoke positivity in the responses from audiences; *Vaccination restriction and contraindication* and *untoward effec*t seem like negative messages, however, openly and accurately reporting side effects of a vaccine could enhance credibility in vaccine safety for audience [40]. The high-promotion individuals were also found to be more susceptible to the factors *credibility* and *official position,* emphasizing the reliability of the message source, possibly because they tend to be more supportive of government actions [41]. Additionally, the attitude of high-prevention individuals was more affected by the messages with *vaccine safety*, *credibility* and *official position*. It has confirmed the concerns about the safety of vaccinations and the reliability of the information source found in previous studies among high-prevention individuals [41,42].

Furthermore, some new findings emerged in Study 2. The attitude of high-promotion individuals was also more influenced by the messages with *vaccine effectiveness* and *risk perception*. *Vaccine effectiveness* messages are usually presented in a gain frame (e.g., “Vaccination reduces the risk of infection by 90%”), which is highly consistent with the preference of high-promotion individuals [43]. The high-promotion individuals were also more susceptible to *risk perception* messages, possibly because vaccination was seen as a means of achieving a normal life in the contextualized COVID-19 infection risk background. The attitude of high-prevention individuals was also more influenced by *responsibility* messages, logically due to the fact that these messages correspond directly to the motivation of highly preventive individuals to comply with social norms.

The audience’s regulatory focus could impact their perception of persuasiveness of the pro-vaccine messages with certain influencing factors. Compared to low-promotion individuals, high-promotion individuals perceived the messages with *vaccine safety*, *vaccination restriction and contraindication*, *untoward effect*, *vaccine effectiveness*, *credibility*, *official position*, and *high standard group* as more persuasive. These results are generally consistent with the results of attitude change, supporting earlier findings that a more persuasive message could lead to greater attitude changes [44]. The message with *high standard group* was found more persuasive for high-promotion individuals and the messages with *vaccine effectiveness* and *interpretability–external attribution* were found more persuasive for high-prevention individuals, but these influences did not appear on the attitude change. One possibility points us to the reason that the persuasiveness of the messages need to reach a certain threshold to induce an attitude change [45].

The influence of regulatory focus on the audiences’ perceived persuasiveness and attitude change seems rarely replicate when measuring their behavioral intention of getting vaccinated. Although the audiences’ traits of high promotion focus or high prevention focus significantly facilitate the effect of several influencing factors on making them feel persuasive or even change their attitudes, similar moderating effect on behavioral intention was only found in the high-promotion audience receiving the message with *vaccine effectiveness.* As previous studies showed, there could be more factors (e.g., the perceived convenience of vaccination) to determine the behavioral intention besides the positive attitude towards the target behavior [46,47]. Our results seem to demonstrate that compared to making people feel persuasive and changing their attitude, there is higher threshold to change their behavior. These results also provide some clues to understand the mechanism underlying the influence of regulatory focus on persuasion. The audiences’ regulatory focus affects their information processing when the pro-vaccine messages incorporate certain influencing factors, making them more likely to comprehend and accept the message. Consequently, they tend to express more positive attitudes. However, this shift in explicit attitude may not translate into the change of behavioral intention. When the pro-vaccine messages emphasize *vaccine effectiveness*, individuals’ high promotion focus not only increased their acceptance of the information but also a significant rise in vaccination intention. This may be because the influence of high promotion focus on information acceptance is strong enough to reach a threshold that alters behavioral intention. Alternatively, it could be that when confronted with such messages, high-promotion individuals activate other cognitive processing pathways, which in turn modify other determinants of behavioral intention, thereby significantly impacting the change in those intentions.

Our results show a clear distinction between high-promotion and high-prevention individuals in vaccination persuasion. High-promotion individuals are primarily motivated by the pursuit of positive outcomes and desires. They are more easily persuaded by information that emphasizes potential benefits, such as the effectiveness of vaccination in preventing disease or the credibility and reliability of the information source. As highlighted in our study, these individuals are highly influenced by messages that portray the safety of vaccines or reinforce trust through official endorsements. They tend to process information in a way that aligns with their goal of achieving positive outcomes, such that they are more likely to respond to cues that appeal to their desire for progress and safety. In contrast, high-prevention individuals are driven by the need to avoid losses and fulfill commitments. They are receptive to messages that emphasize the safety of vaccines and the personal responsibility to get vaccinated. This focus on avoiding harm and adhering to standards makes them more susceptible to the information confirming their concerns about potential risks.

Our findings could also bring some inspirations to the persuading practices in vaccination promotion or even broader field. Although the persuasive effectiveness of the experimental messages in our study was generally higher than the “blank” message as the main effects shown, regardless of participants’ actual trait levels, the different preferences among audiences with different regulatory focus were more deeply and profoundly convinced. As personalized persuasion has been recognized as a highly effective means for influencing attitudes and behaviors [9,48], tailoring messages to align with individual’s regulatory focus would also be instrumental in real life persuasion. Moreover, our study discussed this trait effectiveness in the context of series of specific message contents, providing a wealth of knowledge of how to achieve such trait alignment beyond message framing (Higgins, 2000; Schokker et al., 2010).

Our study focuses on possible changes in individuals’ willingness to be vaccinated that arise from pro-vaccine message. In fact, apart from individuals’ willingness, other factors such as mandatory vaccination could also determine their final vaccine decision. Both theoretically and practically, it has been found that mandatory vaccination, compared to relying solely on individual willingness for voluntary vaccination, can enhance and ensure higher vaccination rates [49–53]. Due to the severity of the COVID-19 pandemic, broad vaccination mandates existed during the practice of vaccine promotion in China. Evidently, these vaccinated individuals cannot be regarded as voluntary vaccinated individuals commonly present in previous studies. They may not genuinely be inclined to receive vaccination and their attitudes towards vaccines usually do not reach a level sufficient to motivate them to voluntarily receive the vaccination [54–56]. Given the scarcity of individuals without contraindications who have not been vaccinated against COVID-19 within the common populace in China, employing the vaccinated individuals in our study is the most authentic approach to representing the state of the public. Exploring what messages and strategies could further enhance the attitudes of these individuals towards vaccination holds practical value for addressing potential future pandemics. These strategies serve to facilitate a shift towards positivity in attitude for individuals unwilling to receive the vaccine, and/or aid in boosting confidence and likelihood of vaccine endorsement for those willing individuals. While it seems not necessary to convince vaccinated individuals to engage in the current wave of vaccination, it is equally important to enhance their confidence about vaccines and increase their willingness to recommend vaccines, which holds significant benefits for increasing vaccine uptake among a broader population and preparing for the potential next wave of vaccinations during the pandemic. Since the significant costs and potential legal challenges of mandatory vaccination [57–59], the aforementioned findings could help in enhancing overall vaccination willingness of public through information dissemination, thereby minimizing the need for mandatory measures. As a personality trait, the impact of regulatory focus on individual’s information preferences exhibits cross-situational stability [60–63], and it is reasonable to infer that the patterns of this impact also hold relevance for persuading individuals in other vaccine persuasion scenarios.

It should also be noted that there are some limitations in this preliminary study. First, while choosing COVID-19 vaccination as the topic in the persuasive research has some obvious advantages, the exceedingly high vaccination rate facilitated by mandatory vaccination also brings restrictions to us. Due to the impracticality of recruiting unvaccinated individuals, our study is based on a sample of those who have undergone mandatory vaccination. As they may receive their first dose about 3 years prior to the study, their responses may be affected by recall bias. This circumstance limits the generalizability of our findings to the unvaccinated population. Our investigation actually reveals the impacts of regulatory focus on the cognitive processing of pro-vaccine messages and potential strategies to enhance the effectiveness of such messages, rather than ensures the compliance of unvaccinated individuals. For future studies when circumstances permit, dedicated examinations of the response of unvaccinated individuals to pro-vaccine messages should be accorded a higher priority. And when applying our research findings to alternative vaccines, a more circumspect analysis with the specific context is preferable. Second, it was conducted in China for the time being, and the findings remain to be further tested in more cross-cultural contexts. It is reasonable to be cautious when generalize our conclusions in different cultural background. Last but not least, the participants in the current study were young and mid-aged adults, while the juveniles and senior citizens were not covered. It could be worthwhile to further examine the impact of regulatory focus in persuasion on the population of other age groups.

## Conclusions

This study investigated the influence of audience’s regulatory focus on the effectiveness of persuasion, in the COVID-19 vaccination scenario. Both promotion focus and prevention focus were found to affect the perceived persuasiveness and attitude change induced by the pro-vaccine messages with certain influencing factors. Compared to low-promotion audiences, high-promotion audiences could generate stronger intention to vaccination by reading the message regarding *vaccine effectiveness*. These results demonstrate the mediating role played by the audience’s personality traits during persuasion processes, and revealed a series of influencing factors in message which were suitable to high-promotion or high-prevention audiences. Our findings not only increase the understanding of how regulatory focus influence the persuasion, but also provide insights to more effective message tailoring and better vaccination promotion towards various groups.

## Supporting information

S1 Appendix The main effect of regulatory focus and influencing factors. (DOCX)

S1 File Raw data used in the analyses of Study 1 and Study 2. (ZIP)
