# Supplementary material for: The influence of audience’s regulatory focus on the persuasive effect of different pro-vaccine messages
Source: PLoS One. 2025 Aug 6;20(8):e0328638. doi: 10.1371/journal.pone.0328638 (PMC12327666; doi:10.1371/journal.pone.0328638)
Supplement: S1 Appendix — (DOCX) [file pone.0328638.s001.docx]

**Appendix.**

**The main effects of the influencing factors.**

**Note.** * represents the influencing factors with a persuasive effect lower than the “blank” message (no influencing factor).

Table 1. The main effects of the influencing factor on attitude changes in Study 1.

| Personality | Influencing factors | *F* | *p* | *η_p_^2^* |
| --- | --- | --- | --- | --- |
| Promotion | Vaccine safety | 206.81 | <0.001 | 0.672 |
|  | Vaccination restriction and contraindication | 53.563 | <0.001 | 0.347 |
|  | Untoward effect | 188.06 | <0.001 | 0.651 |
|  | Vaccine effectiveness | 292.96 | <0.001 | 0.744 |
|  | Credibility | 146.35 | <0.001 | 0.592 |
|  | Official position | 264.00 | <0.001 | 0.723 |
|  | Vaccination certification | 8.412 | 0.005 | 0.077 |
|  | Unofficial position | 153.68 | <0.001 | 0.603 |
|  | Physical benefit * | 8.057 | 0.005 | 0.074 |
|  | Attraction * | 9.86 | 0.002 | 0.089 |
|  | Risk perception | 251.12 | <0.001 | 0.713 |
|  | Responsibility | 124.51 | <0.001 | 0.552 |
|  | High standard group | 8.30 | 0.005 | 0.076 |
|  | Surrounding groups | 54.10 | <0.001 | 0.349 |
|  | Dual role persuasion | 130.05 | <0.001 | 0.563 |
|  | Ingroup pressure | 73.24 | <0.001 | 0.420 |
|  | Social benefits | 1.523 | 0.220 | 0.015 |
|  | Conformity | 2.954 | 0.089 | 0.028 |
|  | Obey | 72.55 | <0.001 | 0.418 |
|  | Interpretability–External attribution | 161.17 | <0.001 | 0.615 |
|  |  |  |  |  |
| Prevention | Vaccine safety | 224.66 | <0.001 | 0.707 |
|  | Vaccination restriction and contraindication | 68.40 | <0.001 | 0.424 |
|  | Untoward effect | 199.00 | <0.001 | 0.681 |
|  | Vaccine effectiveness | 351.30 | <0.001 | 0.791 |
|  | Credibility | 158.48 | <0.001 | 0.630 |
|  | Official position | 311.05 | <0.001 | 0.770 |
|  | Vaccination certification | 10.21 | 0.002 | 0.099 |
|  | Unofficial position | 160.29 | <0.001 | 0.633 |
|  | Physical benefit * | 6.00 | 0.016 | 0.061 |
|  | Attraction * | 4.041 | 0.047 | 0.042 |
|  | Risk perception | 285.69 | <0.001 | 0.754 |
|  | Responsibility | 124.91 | <0.001 | 0.573 |
|  | High standard group | 6.11 | 0.015 | 0.062 |
|  | Surrounding groups | 52.28 | <0.001 | 0.360 |
|  | Dual role persuasion | 130.72 | <0.001 | 0.584 |
|  | Ingroup pressure | 69.60 | <0.001 | 0.428 |
|  | Social benefits | 2.03 | 0.158 | 0.021 |
|  | Conformity | 0.85 | 0.358 | 0.009 |
|  | Obey | 66.42 | <0.001 | 0.417 |
|  | Interpretability–External attribution | 131.713 | <0.001 | 0.586 |

Table 2. The main effects of the influencing factors on attitude changes in Study 2.

| Personality | Influencing factors | *F* | *p* | η_p_^2^ |
| --- | --- | --- | --- | --- |
| Promotion | Vaccine safety | 62.28 | <0.001 | 0.355 |
|  | Vaccination restriction and contraindication | 20.448 | <0.001 | 0.153 |
|  | Untoward effect | 5.21 | 00.024 | 0.044 |
|  | Vaccine effectiveness | 67.46 | <0.001 | 0.374 |
|  | Credibility | 35.92 | <0.001 | 0.241 |
|  | Official position | 58.34 | <0.001 | 0.340 |
|  | Vaccination certification * | 4.25 | 0.042 | 0.036 |
|  | Unofficial position | 1.902 | 0.171 | 0.017 |
|  | Physical benefit * | 57.18 | <0.001 | 0.336 |
|  | Attraction * | 4.07 | 0.042 | 0.035 |
|  | Risk perception | 9.64 | 0.002 | 0.079 |
|  | Responsibility | 36.45 | <0.001 | 0.244 |
|  | High standard group | 5.96 | 0.016 | 0.050 |
|  | Surrounding groups | 0.087 | 0.768 | 0.001 |
|  | Dual role persuasion | 31.32 | <0.001 | 0.217 |
|  | Ingroup pressure * | 37.21 | <0.001 | 0.248 |
|  | Social benefits * | 8.58 | 0.004 | 0.071 |
|  | Conformity | 1.15 | 0.286 | 0.010 |
|  | Obey * | 38.38 | <0.001 | 0.254 |
|  | Interpretability–External attribution | 33.72 | <0.001 | 0.230 |
| Prevention | Vaccine safety | 79.24 | <0.001 | 0.410 |
|  | Vaccination restriction and contraindication | 23.47 | <0.001 | 0.171 |
|  | Untoward effect | 6..48 | 0.012 | 0.054 |
|  | Vaccine effectiveness | 92.82 | <0.001 | 0.449 |
|  | Credibility | 40.94 | <0.001 | 0.264 |
|  | Official position | 56.90 | <0.001 | 0.333 |
|  | Vaccination certification * | 2.18 | 0.142 | 0.019 |
|  | Unofficial position | 0.55 | 0.462 | 0.005 |
|  | Physical benefit * | 74.12 | <0.001 | 0.394 |
|  | Attraction * | 3.21 | 0.076 | 0.027 |
|  | Risk perception | 18.60 | <0.001 | 0.140 |
|  | Responsibility | 42.68 | <0.001 | 0.272 |
|  | High standard group | 5.34 | 0.023 | 0.045 |
|  | Surrounding groups | 1.66 | 0.200 | 0.014 |
|  | Dual role persuasion | 43.87 | <0.001 | 0.278 |
|  | Ingroup pressure * | 23.32 | <0.001 | 0.170 |
|  | Social benefits * | 5.50 | 0.021 | 0.046 |
|  | Conformity | 3.22 | 0.076 | 0.027 |
|  | Obey * | 31.66 | <0.001 | 0.217 |
|  | Interpretability–External attribution | 40.13 | <0.001 | 0.260 |

Table 3. The main effects of the influencing factors on persuasiveness.

| Personality | Influencing factors | *F* | *p* | *η_p_^2^* |
| --- | --- | --- | --- | --- |
| Promotion | Vaccine safety | 78.20 | <0.001 | 0.409 |
|  | Vaccination restriction and contraindication | 27.28 | <0.001 | 0.194 |
|  | Untoward effect | 17.51 | <0.001 | 0.134 |
|  | Vaccine effectiveness | 79.92 | <0.001 | 0.414 |
|  | Credibility | 56.69 | <0.001 | 0.334 |
|  | Official position | 82.23 | <0.001 | 0.421 |
|  | Vaccination certification * | 0.72 | 0.397 | 0.006 |
|  | Unofficial position | 8.03 | 0.005 | 0.066 |
|  | Physical benefit * | 47.86 | <0.001 | 0.298 |
|  | Attraction * | 0.147 | 0.703 | 0.001 |
|  | Risk perception | 29.86 | <0.001 | 0.209 |
|  | Responsibility | 56.302 | <0.001 | 0.333 |
|  | High standard group | 13.58 | <0.001 | 0.107 |
|  | Surrounding groups | 2.56 | 0.112 | 0.022 |
|  | Dual role persuasion | 42.25 | <0.001 | 0.272 |
|  | Ingroup pressure * | 6.78 | 0.010 | 0.057 |
|  | Social benefits * | 1.56 | 0.214 | 0.014 |
|  | Conformity | 9.84 | 0.002 | 0.080 |
|  | Obey * | 8.54 | 0.004 | 0.070 |
|  | Interpretability–External attribution | 59.47 | <0.001 | 0.345 |
| Prevention | Vaccine safety | 71.85 | <0.001 | 0.387 |
|  | Vaccination restriction and contraindication | 22.64 | <0.001 | 0.166 |
|  | Untoward effect | 12.91 | <0.001 | 0.102 |
|  | Vaccine effectiveness | 76.41 | <0.001 | 0.401 |
|  | Credibility | 49.46 | <0.001 | 0.303 |
|  | Official position | 67.38 | <0.001 | 0.371 |
|  | Vaccination certification * | 2.08 | 0.152 | 0.018 |
|  | Unofficial position | 2.38 | 0.126 | 0.020 |
|  | Physical benefit * | 81.11 | <0.001 | 0.416 |
|  | Attraction * | 4.93 | 0.028 | 0.041 |
|  | Risk perception | 21.52 | <0.001 | 0.159 |
|  | Responsibility | 46.12 | <0.001 | 0.288 |
|  | High standard group | 8.54 | 0.004 | 0.070 |
|  | Surrounding groups | 0.79 | 0.376 | 0.007 |
|  | Dual role persuasion | 45.03 | <0.001 | 0.283 |
|  | Ingroup pressure * | 14.10 | <0.001 | 0.110 |
|  | Social benefits * | 3.49 | 0.064 | 0.030 |
|  | Conformity | 4.44 | 0.037 | 0.038 |
|  | Obey * | 16.81 | <0.001 | 0.129 |
|  | Interpretability–External attribution | 47.91 | <0.001 | 0.296 |

Table 4. The main effects of the influencing factors on behavioral intention change.

| Personality | Influencing factors | *F* | *p* | *η_p_^2^* |
| --- | --- | --- | --- | --- |
| Promotion | Vaccine safety | 75.21 | <0.001 | 0.400 |
|  | Vaccination restriction and contraindication | 8.67 | 0.004 | 0.071 |
|  | Untoward effect | 3.61 | 0.060 | 0.031 |
|  | Vaccine effectiveness | 58.62 | <0.001 | 0.342 |
|  | Credibility | 39.97 | <0.001 | 0.261 |
|  | Official position | 57.99 | <0.001 | 0.339 |
|  | Vaccination certification * | 5.28 | 0.023 | 0.045 |
|  | Unofficial position | 0.52 | 0.471 | 0.005 |
|  | Physical benefit * | 50.70 | <0.001 | 0.310 |
|  | Attraction * | 5.51 | 0.021 | 0.046 |
|  | Risk perception | 18.46 | <0.001 | 0.140 |
|  | Responsibility | 40.68 | <0.001 | 0.265 |
|  | High standard group | 8.36 | 0.005 | 0.069 |
|  | Surrounding groups | 0.58 | 0.447 | 0.005 |
|  | Dual role persuasion | 23.07 | <0.001 | 0.170 |
|  | Ingroup pressure * | 22.03 | <0.001 | 0.163 |
|  | Social benefits * | 5.00 | 0.027 | 0.042 |
|  | Conformity | 0.43 | 0.513 | 0.004 |
|  | Obey * | 21.18 | <0.001 | 0.158 |
|  | Interpretability–External attribution | 43.54 | <0.001 | 0.278 |
| Prevention | Vaccine safety | 77.21 | <0.001 | 0.404 |
|  | Vaccination restriction and contraindication | 11.95 | <0.001 | 0.095 |
|  | Untoward effect | 4.83 | 0.030 | 0.041 |
|  | Vaccine effectiveness | 67.24 | <0.001 | 0.371 |
|  | Credibility | 47.23 | <0.001 | 0.293 |
|  | Official position | 57.74 | <0.001 | 0.336 |
|  | Vaccination certification * | 2.286 | 0.133 | 0.020 |
|  | Unofficial position | 1.662 | 0.200 | 0.014 |
|  | Physical benefit * | 64.69 | <0.001 | 0.362 |
|  | Attraction * | 5.98 | 0.016 | 0.050 |
|  | Risk perception | 23.11 | <0.001 | 0.169 |
|  | Responsibility | 46.35 | <0.001 | 0.289 |
|  | High standard group | 10.90 | 0.001 | 0.087 |
|  | Surrounding groups | 2.58 | 0.111 | 0.022 |
|  | Dual role persuasion | 40.17 | <0.001 | 0.261 |
|  | Ingroup pressure * | 19.74 | <0.001 | 0.148 |
|  | Social benefits * | 1.52 | 0.220 | 0.013 |
|  | Conformity | 1.61 | 0.207 | 0.014 |
|  | Obey * | 17.42 | <0.001 | 0.133 |
|  | Interpretability–External attribution | 43.85 | <0.001 | 0.278 |
