# Supplementary material for: The influence of audience’s regulatory focus on the persuasive effect of different pro-vaccine messages
Source: PLoS One. 2025 Aug 6;20(8):e0328638. doi: 10.1371/journal.pone.0328638 (PMC12327666; doi:10.1371/journal.pone.0328638)
Supplement: S1 File — (ZIP) [file pone.0328638.s002.zip › Data Availability Statement.docx]

The data that support the findings of this study are openly available in Dryad at: [URL: <https://datadryad.org/stash/share/xn1wPXCx-oU1qQrgxXbO10G1z_WguRnt-t5e3vDc07s>]
